# Dynamics of epidemic diseases without guaranteed immunity

**DOI:** 10.1186/s13362-021-00101-y

**Published:** 2021-02-22

**Authors:** Kurt Langfeld

**Affiliations:** School of Mathematics, University of Leeds, Leeds, LS2 9JT UK

**Keywords:** Infectious diseases, Coronavirus, SARS-CoV-2, Numerical simulation

## Abstract

The pandemic of Severe Acute Respiratory Syndrome Coronavirus 2 (SARS-CoV-2) suggests a novel type of disease spread dynamics. We here study the case where infected agents recover and only develop immunity if they are continuously infected for some time *τ*. For large *τ*, the disease model is described by a statistical field theory. Hence, the phases of the underlying field theory characterise the disease dynamics: (i) a pandemic phase and (ii) a response regime. The statistical field theory provides an upper bound of the peak rate of infected agents. An effective control strategy needs to aim to keep the disease in the response regime (no ‘second’ wave). The model is tested at the quantitative level using an idealised disease network. The model excellently describes the epidemic spread of the SARS-CoV-2 outbreak in the city of Wuhan, China. We find that only 30% of the recovered agents have developed immunity.

## Introduction

The rapid spread of a disease across a particular region or regions (epidemic) or the global outbreak of a disease (pandemic), see Porta [17], can have a detrimental effect on health systems, on local and global economies including the financial markets and the socio-economic interactions, ranging from the city to the international level. Measures to reduce the pandemic spread include curtailing interactions between infected and uninfected parts of the population, reducing infectiousness or the susceptibility of members of the public, see e.g., Ferguson et al. [5]. The two major strategies governments use to handle an outbreak are to slow down an outbreak (mitigation) or to interrupt the disease spread (suppression). Since each of those interventions bears itself significant risks for the societal and economic well-being, it is crucial to understand the effectiveness of these strategies (or any hybrid of them).

Mathematical methods provide essential input for governmental decision making that aims at controlling the outbreak. Among those are statistical methods, Unkel et al. [22], Becker and Britton [2], deterministic state-space models, Brauer et al. [3] with its prototype developed by Kermack et al. [12], and a variety of complex network models, e.g., Hwang et al. [10], Shirley and Rushton [19]. The different mathematical approaches have different objectives: A significant application of the statistical methods frequently aims at the early detection of disease outbreaks as described by Unkel et al. [22], while modelling either tries to develop a model as realistic as possible for a given outbreak or to design a simplistic model, which, however, reveals some universal truth about the outbreak dynamics.

In the simplest version, the so-called compartmental models (see Kermack et al. [12], Hethcote [9]) consider the fraction of the population which is either susceptible (S), infected (I) or removed (R) from the disease network. Coupled differential equations capture the dynamics of the disease that determine the time dependence of S, I and R. Extensions add more compartments to the Susceptible Infected Removed (SIR) model such as (E) exposed. For example, such an Susceptible Exposed Infected Removed (SEIR) model was used by Lekone and Finkenstädt [15] for a description of the Ebola outbreak in the Democratic Republic of Congo in 1995. Compartmental models have been applied to describe the recent SARS-CoV-2 outbreaks. Selected publications are: Giordano et al. [7], Krishna and Prakash [13], Tagliazucchi et al. [21], Lin et al. [16], Anastassopoulou et al. [1], Wu et al. [23]. For example, the elaborate model from Giordano at al. uses a total of 8 compartments—susceptible (S), infected (I), diagnosed (D), ailing (A), recognized (R), threatened (T), healed (H) and extinct (E)—to describe the **CO**rona **VI**rus **D**isease 20**19** (COVID-19) epidemic in Italy. Compartmental models have been extended by Dureau et al. [4] in order to capture stochastically unknown influences, such as changing behaviours. Such models were recently used to analyse the COVID-19 outbreak in Wuhan by Kucharski et al. [14]. A novel extended epidemic SEIR model, taking into account by a socio-political classification of different interventions, was proposed by Proverbio et al. [18] for assessing the value of several suppression approaches.

Compartmental models address global quantities such as the fraction of susceptible individuals and assume that heuristic rate equations can describe the disease dynamics. In cases of a strongly inhomogeneous (social) network, e.g. taking into account different population densities, the above assumption seems not always be justified. In these cases, spatial disease spread patterns can be described by a stochastic network model with Monte-Carlo simulations a common choice for the simulation.

In this paper, we consider a disease dynamics for which the duration (severity) of the illness depends on the amount of exposure. Using an elementary (social) network, we are looking for universal mechanisms describing a pandemic spread. We will reveal a connection to statistical field theory, enabling us to characterise an outbreak with the tools of critical phenomena. We will discuss the impact of the findings on policies to curb an outbreak and will draw conclusions from the COVID-19 outbreak in Wuhan, Hubei province, China.

## Modelling

### Model basics

Each individual interacts with four ‘neighbours’ of the social network. The disease spread is described as a stochastic process. At each time step (say ‘day’), the probability that an individual gets infected (or recovers) depends on the status of the neighbours in the social network. Here, we only study the simple case of a homogeneous network with four neighbours for each site. We also consider periodic boundary conditions to minimise edge effects.

### Immunity

We study two closely related scenarios.

(i) There is no immunity. Every individual can be reinfected and can recover only to be susceptible again (Susceptible Infected Susceptible (SIS) model).
(ii) Individuals can be reinfected and recover. Only if individuals stay infected for *τ* consecutive days, they are considered *immune*.

In case (ii), the sites of immune individuals are removed from the disease network.

### Disease dynamics

If *x* is a site of the disease network, at every time step the state $u_{x} \in \{0,1\}$ is randomly chosen with probability

1 $$ P(u_{x}) = \frac{1}{{\mathcal{N}}_{x}} \exp \bigl\{ (4 \beta n_{x} + 2 h ) u_{x} \bigr\} ,\quad n_{x} = \sum _{y \in \langle xy \rangle } u_{y} , $$

where $\langle xy \rangle $ is an elementary link on the lattice joining sites *x* and *y* and, hence, *n* is the number of infected neighbours, and ${\mathcal{N}}_{x} =1 + \exp \{ 4 \beta n_{x} + 2 h \} $ is the normalisation. The parameter *h* is linked to the probability to contract the disease from outside the network. In fact, if no-one of the network is infected ($n_{x}=0$, ∀*x*), the probability *p* that any individual contracts the diseases, is connected to *h* by

$$ p = \frac{ \exp \{2h\} }{ 1 + \exp \{ 2h \} } . $$

The parameter *β* describes the *contagiousness* of the disease. The probability that any individual gets infected ($u_{x}=1$) monotonically increases with $4 \beta n_{x} + 2 h$. The parameter *β* hence describes how *sensitive* this probability depends on exposure, i.e., the number $n_{x}$ of infected neighbours.

If the lattice contains *N* individuals (i.e., sites), one time step is said to be completed if we have considered *N* randomly chosen sites for the update.

## The pandemic spread as a critical phenomenon

### Peak infection rate

Scenario (ii) shows the typical time evolution of an epidemic with the infections rate approaching zero for large times due to agents recovering and an increasing number being immune. By contrast, scenario (i) has an asymptotic state independent from the initial state and described by statistical field theory. After the change of variable $z_{x}=2u_{x}-1$, the asymptotic state is described by the partition function of the Ising model, Ising [11], Friedli and Velenik [6]:

$$ Z = \sum_{\{z_{x}=\pm 1\}} \exp \biggl\{ \beta \sum _{\langle xy \rangle } z_{x} z_{y} + H \sum _{x} z_{x} \biggr\} $$

with $H = h + 4 \beta $, which is the well-known partition function for Ising spins *z* in an external magnetic field *H*. The disease dynamics of scenario (i) corresponds to a Markov chain of local updates in the Ising model with Markov time identified as real time.

2 $$ H = 0 , \qquad h(\beta ) = - 4 \beta , \qquad p( \beta ) = \frac{1}{e^{8 \beta } +1 } . $$

**Figure 1:**
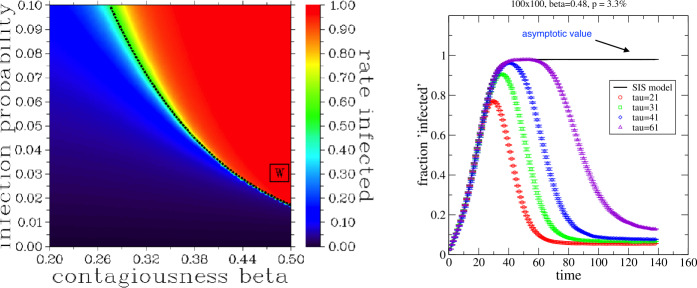
Left: Asymptotic state of the scenario (i): average infection rate as a function of contagiousness *β* and infection probability *p*. Also shown is the critical line $p(\beta )$ in (2) (black dashed line). “W” indicates the parameter set consistent with COVID-19 outbreak in Wuhan. Right: Dynamics of the rate of infected (red bars) compared with the asymptotic value of the field theory (SIS model), which bounds the maximum rate of infected agents

For a vanishing external field *H*, the model shows a critical behaviour with a phase transition at $\beta = \beta _{c} = \ln ( 1 + \sqrt{2} )/2 \approx 0.44$. In the ordered phase for $\beta > \beta _{c}$, a small seed probability $p>0$ triggers an infection rate close to 100 % of the population. The model is in the ‘pandemic’ phase. For $\beta < \beta _{c} $, the model is in the ‘response’ phase, i.e., the infection rate is in response to the seed probability *p*, but no outbreak occurs. The asymptotic infection rate can be calculated using Markov Chain Monte-Carlo (MCMC) methods. Starting, e.g., with no infected agents ($u_{x}=0$ or $z_{x}=-1$), each time step (see Sect. 2) creates one sample of the disease spread. Each disease pattern only depends on that of the previous day, and the sequence of the days form a Markov chain. After some time, which is called ‘thermalisation time’ in statistical physics, the daily infection rate starts to fluctuate around the average, i.e, the asymptotic rate. The asymptotic rate is independent of the simulation details if certain MCMC conditions are satisfied. Among those, ergodicity is easily violated in the pandemic phase for the so-called local update algorithms, most prominently the Metroplis–Hastings one, Hastings [8]. Here, we used the state-of-the-art Swendsen–Wang cluster algorithm, which performs well across both phases, see Swendsen and Wang [20]. Our numerical findings are summarised in Fig. 1, left panel. Curve (2) clearly separates both phases—the pandemic phase and the response regime.

### Immunity

**Figure 2:**
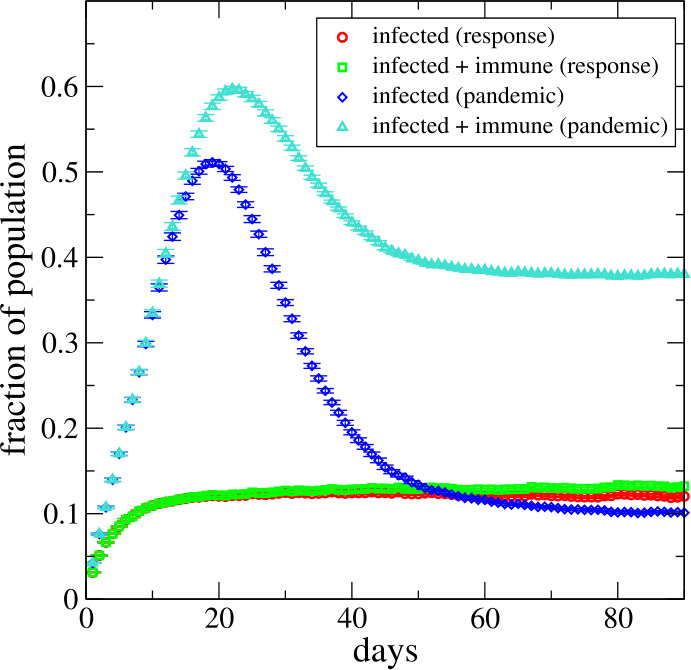
Scenario (ii): rate of infected (red bars) and rate of infected + immune (green bars) for two sets of model parameters: pandemic phase ($p=0.05$, $\beta = 0.41$) and response regime ($p=0.04$, $\beta = 0.38$) both for $\tau =11$

Let us now study scenario (ii), where individuals can develop immunity if they are infected for *τ* consecutive days. For $\tau > t_{\mathrm{th}}$, the peak infection rate is that of the asymptotic state of the corresponding model (i) and, hence, inherits the classification ‘pandemic’ or ‘response’ phase. This is illustrated in Fig. 1, right panel, for the pandemic phase for several values of *τ*. Figure 2 illustrates the vastly different behaviour of the disease spread in the pandemic phase ($\beta = 0.41$, $p =5\%$) and in the response regime ($\beta = 0.38$, $p = 4\%$). Results are for a $N=100 \times 100$ network and $\tau = 11$. Note that the curve for ‘infected + immune’ (‘triangle’ symbol) in the pandemic phase is *not* monotonically increasing with time since the infected individuals can return to ‘susceptible’ state, i.e., not every infected individual becomes immune. Note that in the response regime (‘circle’ and ‘square’ symbol), the ‘pandemic’ peak is absent altogether. However, on the downside, the so-called ‘herd immunity’ slowly develops over time.

### Comparison with data

We stress that the model assumption of a homogeneous (social) network with ‘four neighbours’ is unrealistic. A study of an heterogeneous disease netork is work in porgress. The knowledge of the underlying disease network is essential to make quantitative predictions for e.g. the critical value $\beta _{c}$ of the contagiousness. Here, we adopt a different approach: we assume that qualitative time evolution of bulk quantities such as the fraction of infected individuals is within the grasp of model scenario (ii) and use those as fit functions to determine the model parameters such as *β*, *p* and *τ* by comparison with actual data.

**Figure 3:**
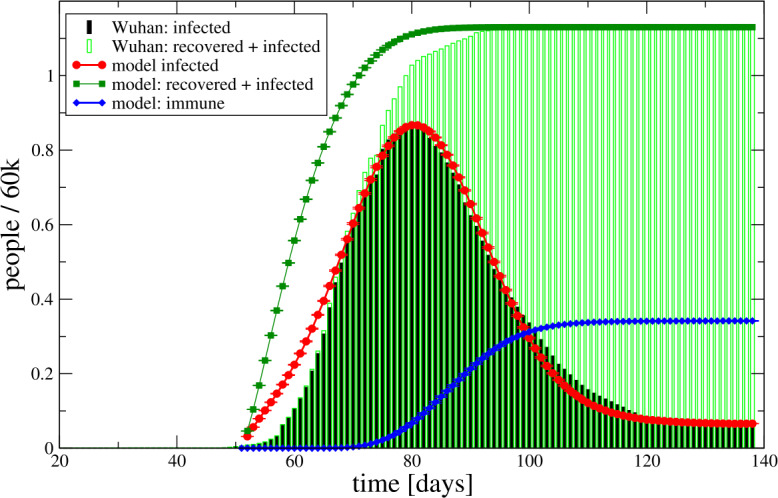
Comparison of scenario (ii) results with actual data from the COVID-19 outbreak in Wuhan

For this study, we used data from the COVID-19 outbreak in 2020 in the city of Wuhan, Hubei province in China, Yu [24] (accessed April 16, 2020). The data of the number of infected individuals shows a jump at day 73 (on the arbitrary time scale) by 40%, which is due to a change in reporting. We assume that the same ‘under-reporting’ has occurred in the days before. Guided by the fact that the probability distribution (the infected rate) is a continuous function, we have corrected the data by multiplying the number of infected (and infected + recovered) by a factor 1.4 for times $t\le 73$. Let $D(t, \tau , \beta , p)$ be the fraction of the population of infected individuals as a function of time *t* and depending on the parameters *τ* (time to develop immunity), *β* (contagiousness) and *p* (seed probability) to get infected. We have calculated $D(t, \tau , \beta , p)$ using a $N=250\times 250$ lattice. We checked that the result is independent of the lattice size in the percentage range for the parameters relevant in this study. If $D_{\mathrm{wuhan}} (t)$ quantifies the measured values for the number of infected in the Wuhan outbreak, we want to approximate these data, i.e.,

$$ D_{\mathrm{wuhan}} (t) \approx N_{\mathrm{pop}} D(t - t_{s}, \tau , \beta , p) $$

with a suitable choice of the parameter $N_{\mathrm{pop}}$, $t_{s}$, *β* and *p*. Since the offset of the time axis in the Wuhan data is arbitrary, we have chosen the shift $t_{s}$ such that the peaks of simulated data and measures data coincide. All other parameters are treated as fit parameters. Those parameters have been obtained by fittng the model to the *infected* data only. Altogether, we find a good agreement with the data for (see Fig. 3):

$$ N_{\mathrm{pop}}\approx 68k, \qquad t_{s} \approx 50, \qquad \tau \approx 21,\qquad \beta \approx 0.48,\qquad p \approx 3.3\% . $$

The results for ‘recovered + infected’ and ‘immune’ are then model predictions. The former can be compared with actual data to gauge the viability of the model.

The model data overshoot the data in the early days of the epidemic spread, which could be related to underreporting due to limited testing capabilities. It is interesting to observe that the curve of the infection rate is asymmetric: the slope of the raise at the beginning is larger than the slope of the decline after the maximum. Also, the number of infected seem to level off at a non-zero value. In the present model, this is explained as follows: with more agents being immune, it is harder for susceptible agents to be continuously infected for time greater or equal *τ* and, thus, to develop immunity. We also find that only about 30% of the infected (and recovered) develop an immunity.

## Conclusions and interpretations

A new type of stochastic disease model is proposed: agents can recover from an infection and are susceptible again. They only develop immunity if their infection lasts longer than a characteristic time *τ*. For $\tau \to \infty $, the infection rate is described by statistical field theory. For finite *τ*, the infection rate of the field theory provides an upper bound of the infection rate of the dynamical model. This opens up the possibility to characterise the disease dynamics in the light of critical phenomena of the underlying field theory: a pandemic spread corresponds to the ordered phase of the field theory, and the critical value for the contagiousness is that for the phase transition. The disease is in controllable response mode if the corresponding field theory is in the disordered phase.

Quantitative results, reported here, are derived with an unrealistic homogeneous disease network for which each agent interacts with four neighbours. Nevertheless, we find that the COVID-19 data of the Wuhan outbreak are well represented. For this case, we find that only 30% of infected develop an immunity.

The heavy tail of the decline of the number of infected, which levels off at non-zero values, is an inherent feature of the model and can be traced back to the fact that agents can be reinfected. In a network with a sizeable portion of immune agents, it is increasingly challenging to develop immunity. If these model assumptions were underpinned by medical investigations, achieving ‘herd immunity’ would be difficult. This should influence the decision to what extent efforts focus on developing a cure or a vaccine.

## Data Availability

The data used to analyse the Wuhan outbreak is publicly available from, Yu [24] (accessed April 16, 2020) or from the author upon request.

## Abbreviations

SARS-CoV-2: Severe Acute Respiratory Syndrome Coronavirus 2
COVID-19: **CO**rona **VI**rus **D**isease 20**19**
SIS model: Susceptible Infected Susceptible model
SIR model: Susceptible Infected Removed model
SEIR model: Susceptible Exposed Infected Removed model
MCMC: Markov Chain Monte-Carlo
